# The advantages of peer review over arbitration for resolving authorship disputes

**DOI:** 10.1186/s41073-019-0071-9

**Published:** 2019-05-30

**Authors:** Zubin Master, Evelyn Tenenbaum

**Affiliations:** 1 Biomedical Ethics Research Program and Center for Regenerative Medicine, Mayo Clinic, 200 First Street, SW, Rochester, MN 55905 USA; 2 Albany Law School, 80 New Scotland Avenue, Albany, NY 12208-3494 USA; 3 Alden March Bioethics Institute, Albany Medical College, 47 New Scotland Avenue, MC 153, Albany, NY 12208-3478 USA

**Keywords:** Authorship, Authorship disagreement, Arbitration, Peer review, Research ethics consultation

## Abstract

A recent commentary argued for arbitration to resolve authorship disputes within academic research settings explaining that current mechanisms to resolve conflicts result in unclear outcomes and institutional power vested in senior investigators could compromise fairness. We argue here that arbitration is not a suitable means to resolve disputes among researchers in academia because it remains unclear who will assume the costs of arbitration, the rules of evidence do not apply to arbitration, and decisions are binding and very difficult to appeal. Instead of arbitration, we advocate for peer-based approaches involving a peer review committee and research ethics consultation to help resolve authorship disagreements. We describe the composition of an institutional peer review committee to address authorship disputes. Both of these mechanisms are found, or can be formed, within academic institutions and offer several advantages to researchers who are likely to shy away from legalistic processes and gravitate towards those handled by their peers. Peer-based approaches are cheaper than arbitration and the experts involved have knowledge about academic publishing and the culture of research in the specific field. Decisions by knowledgeable and neutral experts could reduce bias, have greater authority, and could be appealed. Not only can peer-based approaches be leveraged to resolve authorship disagreements, but they may also enhance collegiality and promote a healthy team environment.

## Background

Zen Faulkes wrote a provocative piece outlining that arbitration may be a suitable mechanism to resolve disputes among researchers employed in academia who cannot work out their differences over authorship [1]. While not a completely new idea [2], Faulkes explains that despite the adoption of authorship guidance, there are few proposals on how to resolve authorship issues—something we agree on. Yet, in several instances, he compares arbitration with peer review based systems explaining that arbitration is similar to research ethics consultation services or committee review. It is not. Arbitration generally involves having representation and an arbitrator. There are costs associated with this process, and it is unclear who should foot the bill. Also, while certainly faster and cheaper than litigation, the rules of evidence do not apply in arbitration and decisions made by arbitrators are usually final. We applaud the paper for addressing this crucial topic impacting all of us in academia, but take issue that arbitration would actually be a frequently used mechanism. Instead, we promote resolution approaches based on peer review—approaches that are also likely more palatable to academic researchers. We argue that extant peer-based mechanisms of research ethics consultation services or an institutional peer review committee would be able to address authorship disputes without the shortcomings of arbitration and likely provide a means to resolve authorship disagreements collegially. While there is some literature on the function of research ethics consultation services, we go further to describe the function and composition of a proposed institutional peer review committee to help resolve authorship disputes.

## Main text

### The limits of arbitration

It is well understood that arbitration is usually cheaper and faster than litigation, but arbitration can have significant costs. For consumer arbitration, the American Arbitration Association charges businesses a filing fee of $300 for a single arbitrator, a $1400 case management fee, and $1500 for compensating the arbitrator [3]. In international settings, the costs depend on the amount of the claim. Claims less than $75,000 have initial and final filing fees of $1000 with an extra 10% for additional separately represented parties [4]. Faulkes assumes that journals or publishers might cover arbitration costs as a mark of excellence. While publishers are interested in authorship disputes being resolved swiftly and collegially, they will be reluctant to pay for arbitration services and having a badge of recognition is unlikely sufficient motivation. Funding agencies might cover these costs, but they have no policies permitting the use of funds for arbitration. It also becomes complicated when multiple funders with multiple Principal Investigators are involved in an authorship disagreement. Likely, bearing costs will fall on institutions and institutions already have suitable mechanisms in place to resolve authorship disputes.

Another limit of arbitration is that the state and federal rules of evidence do not apply [5]. This means that information that would not be admitted in a court of law can be accepted by an arbitrator. For example, documents may be introduced without the testimony of witnesses, depriving authors of the opportunity to cross-examine or adequately question the evidence submitted. Discovery may also be limited, which can deprive authors of access to information essential to prove their case.

In addition, arbitrators’ decisions are generally final and binding; they are very difficult to appeal [6]. This is true even if the arbitrator makes a glaring mistake on the law/policy, fails to understand the subtleties relating to the dispute, does not carefully and thoroughly review the documents, or is unfairly biased towards one party. The right to appeal would protect authors by giving them the possibility of a new review by an impartial panel of judges.

### A more plausible solution

As Faulkes compares arbitration to research ethics consultation services and peer review committees, why not just have these processes help resolve authorship conflicts. An academic peer review system makes sense because most research ethics issues, including human and animal subject violations, conflicts of interest and industry relations, and research misconduct allegations are handled within academic institutions. Despite the flaws inherent to peer review, it is still a recognized and generally accepted approach used pervasively in academia.

Currently, research ethics consultants provide information, help navigate researchers, increase sensitivity among researchers, and can help resolve issues related to research, including managing and mediating authorship disagreements [7]. Research ethics consultations can prevent the escalation of authorship disagreements, but are unlikely to make clear judgments about authorship inclusion or order because of their largely informational and navigation functions [8]. On the other hand, an institutional peer review committee could not only help review a case, but also make decisions that all authors could agree to uphold [9].

Little is known about the composition of institutional peer review committees surrounding issues of research integrity [10], much of which centers on investigating research misconduct. But professional peer review bodies that exist in medical and dental practice aim to enhance care and ensure professionalism [11–13] and evaluate academic promotion and research grants [14]. The structure of such committees offers guidance on the potential composition of an institutional peer review committee to address authorship disputes.

Institutional peer review committees addressing issues of research integrity specific to authorship should include scientists knowledgeable in the area of research with substantial publication experience, and who have collaborated with researchers external to the institution. These faculty scientists should range in seniority and, to avoid bias, cannot be from the same department(s) as the research team with the authorship dispute. Such scientists should understand institutional culture, but remain open-minded. Depending on the size and diversity of the institution, having a scientist who is new, or external, to the institution could provide fresh or alternative perspectives. The committee should also consist of an ethics expert with knowledge in research integrity and authorship ethics policies and practices. Possible additional members might include research ethics consultants, a Research Integrity Officer, or other ethics experts within the institution. If appropriate research or ethics expertise is not found within the institution, external members can always be solicited. If possible, either one of the scientific or ethics members of the committee, or another individual, with experience handling disagreements and disputes, and with training in cultural competency, such as a coach, might be helpful. Lastly, a trainee representative with publication experience should be on the committee to represent and provide a trainee’s perspective because trainees have less power and their voice may otherwise go unnoticed.

None of the committee members should have any conflicts of interest with the research team(s) with the authorship dispute. Committees can be convened on an ad hoc basis, but depending on institutional needs, it may be better to have a standing committee with rotating members with fixed-term appointments in order to maintain corporate knowledge and bring in appropriate expertise depending on the nature of the dispute. Committees should be a manageable size consisting of about five members and a seasoned chair who is known for being fair and unbiased. Committees should render a final decision and produce a report of the deliberation on the authorship case. We feel that recommendations regarding reprimands is beyond the scope of this committee and would be better handled by Human Resources if and when needed.

With an institutional peer review committee making decisions, the interested parties could present their arguments and receive a full review of the issues. In general, this should resolve the matter, but in the event there is an arbitrary decision, bias, or failure to consider some salient factors, the option to appeal the committee’s decision or move to binding arbitration remains.

A peer review based system has two major advantages. First, a peer review system for resolving authorship disputes would likely be cheaper than arbitration as it involves the time of academics and the costs would be absorbed by institutions. Faulkes mentions that such systems are restricted to researchers in a single institution, but this does not have to be the case. Research ethics consultants can work not only with researchers at their institution, but also externally to resolve a host of research ethics and integrity issues. For example, authorship disputes involving one or more researchers who left an institution before a manuscript was published have been handled by research ethics consultations. Similarly, institutional peer review committees can be formulated with members from other research institutions for authorship disagreements involving parties from different institutions. Here, a balance would be needed to maintain a modest committee size, but have fair representation among institutions. In addition, the costs of setting up and managing an institutional peer review committee would not be onerous and could be lessened by making service on the panel prestigious and an institutional service commitment. Among well-resourced institutions, relief from teaching duties or the requirement of bringing in salary support from grants would be strong incentives for researchers to undertake such service commitments.

Second, not only will a peer review system have researchers who are at arms-length with the research team but, unlike judges and arbitrators, they will have special expertise and knowledge about academic publishing and the culture of research in that specific field and institution. Having knowledge and experience in authorship ethics could facilitate a sense of authority over the subject especially when addressing the subtleties of publishing. This is important because very few researchers (9% of 4043 respondents) report having substantial knowledge in publication ethics [15]. Moreover, a discussion of authorship issues with a committee or a consultant could facilitate a reciprocal understanding with involved parties and avoid escalating the situation and creating an uncomfortable environment. Decisions made by expert panels may be given extra weight because they are generally accepted by the academic community.

## Conclusions

Addressing research ethics issues by peer-based mechanisms will uphold scientific autonomy, be more cost-effective, and likely resolve issues in a peaceful way thereby creating a more harmonious atmosphere. It is quite unlikely that arbitration to resolve authorship feuds will be entertained by most academic researchers who tend to gravitate away from legalistic procedures. Authorship issues can arise for many reasons, but cultivating a respectful team environment is likely to lead to a healthier environment and more productive research [16]. Recognizing the human dimensions of authorship and publication ethics can best be facilitated through scientific peers well versed in authorship ethics and the culture of research.

## Pluralism in authorship dispute resolution: reply to Master and Tenenbaum

Zen Faulkes

Department of Biology, The University of Texas Rio Grande Valley, 1201 West University Drive, Edinburg, TX, 78539, USA

Correspondence: zen.faulkes@utrgv.edu

### Abstract

Research communities currently have few mechanisms to address authorship disputes, leading to the question of whether alternative dispute resolution could be used to resolve such disputes. Master and Tenenbaum critique one form of alternative dispute resolution (arbitration) and propose a peer review model of dispute resolution. Their comments highlight that dispute resolution can take many forms other than arbitration and that research communities might profitably use a diverse set of methods to help resolve authorship disputes.

### Background

Previously, I argued that authorship disputes are too often left entirely for the authors to solve, and such “hands off” approaches to dispute resolution have corrosive effects on research communities [1]. This creates an ethical imperative for research communities to adopt some form of dispute resolution, of which there are several models. Master and Tenenbaum (above) examine the possible complications of using arbitration to resolve authorship disputes and suggest a peer review model of dispute resolution instead.

### Main text

In responding to “Resolving authorship disputes by mediation and arbitration” [1], Master and Tenenbaum (above) focus almost exclusively on arbitration. Arbitration is not the only form of alternative dispute resolution [17] that is discussed in the original article [1]. Some points raised by Master and Tenenbaum would not apply to all forms of alternative dispute resolution. For example, whether there is an appeals process is more relevant to arbitration than it is to mediation. Concerns about arbitration specifically should not rule out considering how other forms of alternative dispute resolution might be used to resolve authorship disputes.

Master and Tenenbaum (above) are concerned about the financial costs of arbitration, particularly if administered through journals [1]. The financial costs of alternative dispute resolution would be immediate, direct, and possibly substantial, and any financial benefits would be delayed, indirect, and possibly small. When performing a cost/benefit analysis, however, there can be costs and benefits that are not easily counted using money. There is no accounting for the cost of lost opportunities or the benefits of having high ethical standards. Accountants might view quality assurance on airplanes as a sunk cost that cannot be recovered by the sale of the plane, but catastrophic failures (e.g., the plane crashes) make intangible benefits apparent. Moreover, absolute costs need to be considered within a larger context of budgets. Some academic publishers are non-profit or simply small, and the relative cost of arbitration may be prohibitive. But many academic publishers are highly profitable [18, 19], and the relative cost of arbitration may be minimal and completely feasible for them.

Master and Tenenbaum predict that “for-profit publishers are not interested in resolving disputes and having a badge of recognition is unlikely sufficient motivation” (above). Somewhat contrary to this prediction, several journals have hired research integrity officers and/or created research integrity teams [20]. Their responsibility includes many ethical issues, including authorship disputes. This suggests that journals see value in maintaining high ethical standards and is consistent with the suggestion in the original article that journals be involved with dispute resolution [1].

Master and Tenenbaum (above) mention that arbitration rarely permits appeals. The pros of appeals are that they allow revisiting unfair decisions, but the cons are that they can add time, cost, and complexity to resolving disputes. In courts, appeal processes tend to favor individuals with the most power [21], which is an important consideration given that authorship disputes can involve deep power differences [1].

The model of “peer review” resolution advanced by Master and Tenenbaum might be considered another type of alternative dispute resolution, since it is not a legal process. As with any initial proposal, there are unanswered questions about how such a model would be implemented (e.g., what institutions would peers would be drawn from?). Their peer review model provides another useful option for authors, editors, and others involved in scientific publishing to consider.

### Conclusions

Just as there is diversity in scientific publishing practices, there is room for research communities to adopt a pluralistic approach to dispute resolution. Importantly, both Master and Tannebaum and I seem to agree that the status quo on authorship disputes is problematic and that research communities should not walk away from disputes.
